# High Frequencies of Senescent and Exhausted Natural Killer Cells and SARS-CoV-2-Specific Memory CD8^+^ T Cells Distinguish Critically Ill from Asymptomatic Unvaccinated COVID-19 Patients

**DOI:** 10.3390/v18090940

**Published:** 2026-08-28

**Authors:** Ruchi Srivastava, Swayam Prakash, Sweta Karan, Lbachir BenMohamed

**Affiliations:** 1 Laboratory of Cellular and Molecular Immunology, Gavin Herbert Eye Institute, School of Medicine, University of California Irvine, Irvine, CA 92697, USA; ruchi.srivastava@uci.edu (R.S.); fswayamp@hs.uci.edu (S.P.); skaran1@hs.uci.edu (S.K.); 2 Institute for Immunology, School of Medicine, University of California Irvine, Irvine, CA 92697, USA; 3 TechImmune, Inc., Irvine, CA 92617, USA

**Keywords:** SARS-CoV-2, COVID-19, senescence, NK cells, CD8^+^ T cells

## Abstract

COVID-19 disease severity in unvaccinated individuals spans a striking continuum from complete absence of symptoms to life-threatening critical illness, yet the immunological determinants driving this divergence remain poorly understood. While Natural Killer (NK) cells and CD4^+^/CD8^+^ T cells mount distinct responses against SARS-CoV-2, the phenotypic and functional signatures distinguishing protective immunity from pathogenic immune dysfunction have yet to be defined. In the present study, we present a comparative analysis of NK cell and SARS-CoV-2-specific T cell immunity in unvaccinated symptomatic (SYMP) versus asymptomatic (ASYMP) COVID-19 patients. Using multicolor flow cytometry, we interrogated key immune checkpoints, the senescence marker CD57, differentiation status (CD45RA/CCR7), exhaustion marker PD-1, and activation markers HLA-DR and CD38, across NK and T cell subsets from SYMP patients, ASYMP patients, and Healthy Donors (HD). SYMP COVID-19 patients harbor significantly elevated co-expression of exhaustion and senescence markers on both NK and T cells relative to ASYMP patients and HD controls. Within the T cell compartment, SYMP patients exhibit broad dysregulation across naïve, central memory, effector memory, and terminally differentiated effector memory subsets, with CD57 enriched most prominently on CD8^+^ T_EM_ and CD8^+^ T_EMRA_ populations. While cytokine profiles did not significantly differ between ASYMP and SYMP patients, both groups showed markedly elevated pro-inflammatory TNF-α, IFN-γ, IL-6, IL-8, and IL-17 compared to HD controls, underscoring systemic immune activation as a hallmark of SARS-CoV-2 infection regardless of clinical severity. Taken together, these findings suggest that NK and T cell exhaustion and senescence are correlates of severe COVID-19, highlighting potential targets for future mechanistic and therapeutic investigation.

## 1. Introduction

Severe acute respiratory syndrome coronavirus 2 (SARS-CoV-2), a β-coronavirus first identified in Wuhan, China, in late 2019, rapidly evolved into one of the most consequential infectious disease crises in modern history [1]. As of June 2026, more than 704 million confirmed COVID-19 cases and approximately 7.1 million confirmed deaths have been documented globally [2,3,4,5,6,7,8,9]. Despite the scale of this ongoing pandemic, one of its most perplexing and clinically critical features remains incompletely understood: why does infection with the same pathogen produce a spectrum of outcomes ranging from the complete absence of symptoms to fatal respiratory failure?

COVID-19 clinical severity spans a striking continuum. While 80–85% of newly infected unvaccinated individuals remain asymptomatic (ASYMP, i.e., testing positive for SARS-CoV-2 yet experiencing no clinical disease), a subset, particularly the elderly and immunocompromised, develop severe symptomatic (SYMP) disease characterized by progressive pulmonary inflammation, cytokine storm, acute respiratory distress syndrome (ARDS), and death [10,11,12,13,14,15]. The spectrum of symptomatic to severely symptomatic presentations of COVID-19 may represent the balance between innate and adaptive immune responses: in asymptomatic patients, a prompt and adequate adaptive immune response quells infection, whereas in those with severe symptoms, an inadequate immunopathological response leads to a runaway cytokine cascade fueled by ongoing viral replication. Yet, the precise cellular immunological determinants that separate protection from pathology, and particularly the roles of Natural Killer (NK) cells and SARS-CoV-2-specific T cells, remain to be fully defined.

Innate and adaptive immune responses are both essential for effective antiviral immunity [9,16]. NK cells serve as first responders during acute viral infection, providing immediate cytotoxic surveillance before antigen-specific T cells can be mobilized, while effector CD4^+^ helper and cytotoxic CD8^+^ T cells are indispensable for durable viral clearance and long-term immune memory [6,9,17]. Asymptomatic individuals tend to establish a more effective and protective immune phenotype by maintaining functional T cells and NK cells alongside a relatively balanced immune response, a stark contrast to the immune dysregulation observed in severe COVID-19 patients [18,19]. Importantly, SARS-CoV-2-specific T cell frequencies are similar between asymptomatic and symptomatic severely ill COVID-19 patients, yet asymptomatic individuals show increased IFN-γ and IL-2 production, indicating that the quality, not the quantity, of the antiviral T cell response governs clinical outcome [17,20]. Asymptomatic individuals mount potent virus-specific CD4^+^ Th1 and cytotoxic CD8^+^ T cell responses, whereas patients with moderate to severe COVID-19 disease show minimal induction of these protective cellular responses, despite vigorous humoral responses, revealing a strikingly dichotomous and incomplete adaptive immune landscape across disease severity [2,5,21].

A key mechanism underlying this immune failure in severely ill COVID-19 patients is the progressive dysfunction of both NK and T cell compartments through exhaustion and senescence [9,16]. T cell exhaustion is a terminal state of immune dysfunction characterized by impaired proliferation and effector functions, diminished cytotoxic function and cytokine secretion, and sustained expression of inhibitory receptors. Increasing evidence links exhausted T cell phenotypes with poor clinical outcomes in COVID-19, including severe disease, delayed viral clearance, and persistent symptoms. This exhausted state is marked by upregulation of inhibitory checkpoint receptors, including PD-1, TIM-3, LAG-3, CTLA-4, NKG2A, and CD39 [2,5]. Parallel dysfunction occurs within the innate compartment: NK cells from critically ill COVID-19 patients are highly activated yet exhausted, exhibiting poor cytotoxic function and impaired cytokine production upon stimulation. The mechanisms that orchestrate immune cell dysfunction and virus interaction in severe COVID-19 remain unknown. Some studies link adaptive immune cell exhaustion to epigenetic regulation of inhibitory immune checkpoint receptors, while other studies link over-activation of T cells to enhanced uncontrollable alveolar inflammation and cytokine storm [9,16].

Compounding this exhaustion is the emergence of cellular senescence, a distinct but overlapping state of terminal immune dysfunction. CD57, a key marker of replicative senescence, identifies terminally differentiated lymphocytes with shortened telomeres, diminished proliferative capacity, and an inability to mount effective recall responses [6,9,17]. Repeated antigen stimulation during chronic or severe viral infection drives upregulation of CD57 on both T cells and NK cells, marking cells that, while potentially cytotoxic through CD16 signaling, are unresponsive to cytokine activation and incapable of sustained antiviral surveillance [2,3,21,22]. Immunosenescence thus represents a critical bottleneck in viral clearance: an unbalanced innate and adaptive immune recovery following SARS-CoV-2 infection can lead to a senescent phenotype with impaired CD8^+^ T cell function, perpetuating viral persistence and driving the inflammatory environment characteristic of critical illness. Despite these insights, the phenotypic and functional signatures of senescent and exhausted NK and T cell subsets, and how they differ across the asymptomatic-to-critical severity spectrum in unvaccinated individuals, remain incompletely characterized [2,19,23,24].

To address this gap, we present a systematic comparative analysis of the frequency, phenotypic, and functional characteristics of NK cells and SARS-CoV-2-specific CD4^+^ and CD8^+^ T cells in unvaccinated symptomatic (SYMP) and asymptomatic (ASYMP) COVID-19 patients and healthy donors (HD). We interrogated key markers of immune senescence (CD57), differentiation status (CD45RA/CCR7), exhaustion (PD-1), and activation (HLA-DR, CD38) across NK and T cell subsets, alongside plasma cytokine profiling. Our findings reveal a convergence of NK and T cell exhaustion and senescence that distinguishes critically ill from asymptomatic COVID-19 patients, with senescent CD8^+^ T cell effector populations representing a prominent immunological correlate of severe COVID-19 disease that warrants further investigation.

## 2. Materials and Methods

### 2.1. Human Study Population

Twenty unvaccinated COVID-19 patients (ten Asymptomatic and ten Symptomatic) and ten unvaccinated healthy donors, who had never been exposed to SARS-CoV-2, were enrolled in this study (Table 1). Forty percent were Caucasian, and 60% were non-Caucasian. Forty percent were females, and 60% were males, with an age range of 21–67 years (median 39). None of the symptomatic patients were on antiviral or anti-inflammatory drug treatments during blood sample collection. No COVID-19 patient or healthy donor had received a COVID-19 vaccination. Medical history and laboratory data were retrospectively reviewed. The most relevant patient characteristics of the unvaccinated symptomatic and asymptomatic COVID-19 patients and the unvaccinated unexposed healthy individuals concerning age, gender, HLA-A*02:01, and HLA-DR distribution, COVID-19 disease severity, comorbidity, and biochemical parameters are detailed in Table 1. COVID-19 was microbiologically documented by RT-PCR in nasopharyngeal specimens collected before recruitment. Asymptomatic individuals presented no symptoms at all despite being documented as being infected with SARS-CoV-2. Symptomatic individuals have critical illness, as defined by the Centers for Disease Control and Prevention (CDC). The average number of days between the report of their first symptoms and the blood sample collection was 4.8 days. PBMC and plasma specimens were obtained by centrifugation of whole blood ethylenediaminetetraacetic acid tubes, cryopreserved at −80 °C, and retrieved for analyses within 15 days after collection. All clinical investigations in this study were conducted according to the Declaration of Helsinki principles. All subjects were enrolled at the University of California, Irvine, under approved Institutional Review Board protocols (IRB#-2020-5779). Written informed consent was received from all participants before inclusion.

### 2.2. HLA-A2 Typing

The HLA-A2 status was confirmed by staining PBMCs with 2 μL of anti-HLA-A2 mAb (clone BB7.2) (BD Pharmingen, Franklin Lakes, NJ, USA) at 4 °C for 30 min. The cells were washed and analyzed by flow cytometry using an LSRII (Becton Dickinson, Franklin Lakes, NJ, USA). The acquired data were analyzed with FlowJo software (BD Biosciences, San Jose, CA, USA). HLA-A*02:01 positivity was not used as an a priori inclusion criterion during recruitment; rather, HLA-A2 typing was performed on PBMCs from all enrolled participants after enrollment, as described above. Only participants confirmed to be HLA-A*02:01-positive by this assay were subsequently included in the tetramer-based antigen-specific analyses reported in this study.

### 2.3. Tetramer/Peptide Staining

Fresh PBMCs were analyzed for the frequency of CD8^+^ T cells recognizing the SARS-CoV-2 peptide/tetramer complexes, as we previously described in [25]. The cells were incubated with SARS-CoV-2 peptide/tetramer complex for 30–45 min at 37 °C. The cell preparations were then washed with FACS buffer and stained with FITC-conjugated anti-human CD8 mAb (BD Pharmingen), as described [26]. Finally, the cells were washed and fixed with 1% paraformaldehyde in PBS and subsequently acquired on a BD LSRII. Data were analyzed using FlowJo version 9.5.6 (Tree Star).

### 2.4. Human Peripheral Blood Mononuclear Cells (PBMC) Isolation

SARS-COV-2 positive individuals were recruited at the UC Irvine Medical Center. Between 40 and 50 mL of blood was drawn into yellow-top Vacutainer^®^ Tubes (Becton Dickinson), as described [26]. The plasma samples were isolated and stored at −80 °C for the detection of various cytokines using Luminex (Luminex Corporation, Austin, TX, USA). PBMCs were isolated by gradient centrifugation using a leukocyte separation medium (Life Sciences, Tewksbury, MA, USA). The cells were then washed in PBS and re-suspended in a complete culture medium consisting of RPMI1640, 10% FBS (Bio-Products, Woodland, CA, USA), supplemented with 1x penicillin/streptomycin/L-glutamine, 1x sodium pyruvate, 1x non-essential amino acids, and 50 μM of 2-mercaptoethanol (Life Technologies, Rockville, MD, USA). For future testing, freshly isolated PBMCs were also cryopreserved in 90% FCS and 10% DMSO in liquid nitrogen.

### 2.5. Human T Cells Flow Cytometry Assays

The following anti-human antibodies were used for the flow cytometry assays: CD3 Percp, CD8 APC-Cy7, CD57 PE-Cy7, PD-1 A647, CD45RA FITC, CCR7 BV786, HLA-DR BUV385, CD38 A700, and CD56 APC (BioLegend, San Diego, CA, USA), as described [26]. For surface staining, mAbs against cell markers were added to a total of 1 × 10^6^ cells in 1X PBS containing 1% FBS and 0.1% sodium azide (FACS buffer) for 45 min at 4 °C. After washing with FACS buffer, cells were permeabilized for 20 min on ice using the Cytofix/Cytoperm Kit (BD Biosciences) and then washed twice with Perm/Wash Buffer (BD Biosciences). Cells were washed with Perm/Wash and FACS Buffer and fixed in PBS containing 1% paraformaldehyde (Sigma-Aldrich, St. Louis, MO, USA), as described [26]. For each sample, 100,000 total events were acquired on the BD LSRII. Ab capture beads (BD Biosciences) were used as individual compensation tubes for each fluorophore in the experiment. We used fluorescence minus controls for each fluorophore to define positive and negative populations when initially developing staining protocols. In addition, we further optimized gating by examining known negative cell populations for background expression levels. The gating strategy was similar to that used in our previous work [27]. Briefly, we gated on single cells, dump cells, viable cells (Aqua Blue), lymphocytes, CD3^+^ cells, and human epitope-specific CD8^+^ T cells using SARS-CoV-2-specific tetramers, as described [26].

**Figure 1:**
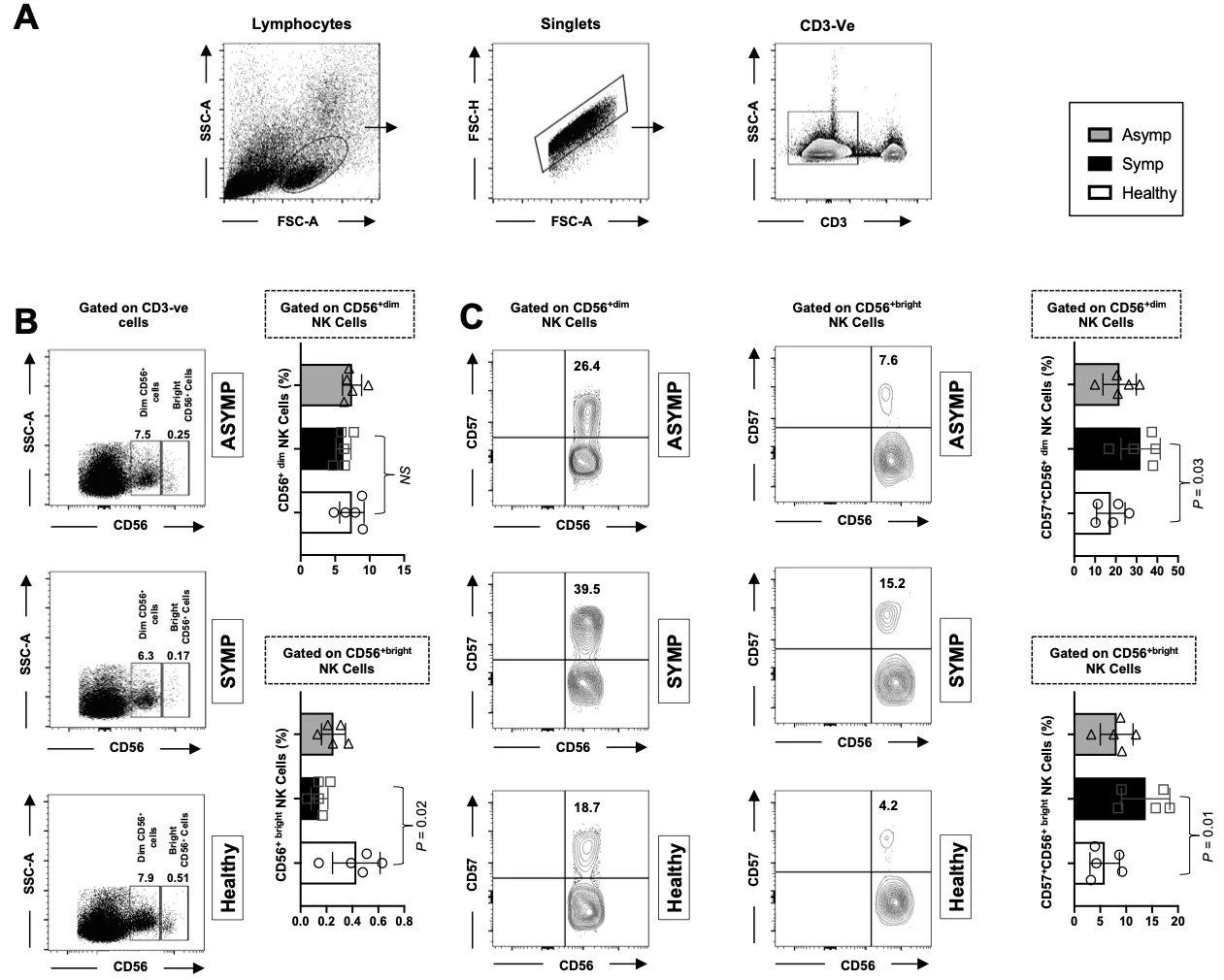
Composition of NK cell subsets in COVID-19 SYMP individuals shows a decreased CD56^bright^ NK cell population and a higher frequency of mature/terminally differentiated NK cells (CD57^+^) than in healthy individuals: a gating strategy for defining the NK cell population is shown using FACS. (**A**) The lymphocyte populations were gated using forward scatter (FSC) and side scatter (SSC). Singlets were gated after gating the lymphocyte population, and NK cells were defined as CD3^−^ CD56^+^ cells. The NK cell population was further categorized into CD56^dim^ and CD56^bright^ cells, and their relative frequency of senescence (CD57^+^) was evaluated. (**B**) Representative FACS data of the frequencies of CD56^dim^ NK cells and CD56^bright^ NK cells detected in PBMCs from COVID-19 ASYMP individuals, SYMP individuals, and healthy donors (**left** panel). Average frequencies of PBMC-derived CD56^dim^ NK cells (**top**) and CD56^bright^ NK cells (**bottom**) were detected from ASYMP, SYMP, and healthy donors (**right** panel). (**C**) Representative FACS data of the frequencies of CD57 gated on CD56^dim^ NK cells and the frequencies of CD57 gated on CD56^bright^ NK cells detected in PBMCs from a COVID-19 ASYMP individual, SYMP individual, and healthy donor (**left** panel). Average frequencies of PBMCs-derived CD56^dim^ NK cells (**top**) and CD56^bright^ NK cells (**bottom**) were detected from ASYMP, SYMP, and healthy donors (**right** panel). The results are representative of two independent experiments on each individual. Samples from ten ASYMP and ten SYMP patients were used, and the right columns show representative results from five ASYMP and five SYMP patients from two experiments run independently and consecutively. The indicated *p* values, calculated using an unpaired *t*-test, show statistical significance between SYMP and healthy donors.

The gating strategy was similar to that used in our previous work [26] and is shown in full for a representative donor in Supplementary Figure S1. Briefly, lymphocytes were first identified by forward scatter (FSC-A) versus side scatter (SSC-A); single cells were resolved from doublets using FSC-H versus FSC-A; a dump/viability gate (Aqua Blue viability dye together with a dump channel) was applied to exclude dead cells and non-target lineages; CD3^+^ cells were gated for T cell analyses, and CD3^−^CD56^+^ cells were gated for NK cell analyses (Figure 1A); downstream analyses were performed within the CD3^+^CD8^+^ or CD3^+^CD4^+^ T cell gates, or the CD56^dim^/CD56^bright^ NK cell subgates, followed by identification of SARS-CoV-2 peptide/tetramer-positive cells within the CD8^+^ T cell gate. Positive gates for phenotypic markers (CD57, PD-1, CD38, HLA-DR) were placed using fluorescence-minus-one (FMO) controls for each fluorophore, with the gate boundary set at the point where FMO-control events comprised ≤0.1–0.5% of the parent population; this threshold was applied uniformly across all samples analyzed within a given experiment. Known biologically negative populations within each sample (e.g., non-tetramer-binding CD8^+^ T cells for tetramer positivity) served as an additional, sample-intrinsic reference to confirm gate placement and control for day-to-day and donor-to-donor variability in background staining. Data analysis was performed using FlowJo version 9.9.4 (TreeStar, Ashland, OR, USA). Statistical analyses were done using GraphPad Prism version 5 (La Jolla, CA, USA).

### 2.6. Statistical Analyses

Data for each assay were compared by analysis of variance (ANOVA) and Student’s *t*-test using GraphPad Prism version 5.03. ANOVA and multiple comparison procedures identified differences between the groups, as we previously described in [28]. Data are expressed as the mean ± SD. Results were considered statistically significant at *p* < 0.05.

## 3. Results

### 3.1. Composition of NK Cell Subsets in COVID-19 SYMP Patients Shows a Decreased CD56^bright^ NK Cell Population and a Higher Frequency of Mature/Terminally Differentiated NK Cells (CD57^+^) Compared to Healthy Individuals

NK cells are a subset of innate immune lymphocytes composing 5% to 20% of PBMCs in humans and play an important role in the defense against viral infections. These cells are reduced in number, but less consistently than T cells, particularly in severely sick patients. Therefore, we first investigated the phenotypic status of NK cells in SARS-CoV-2 positive asymptomatic (ASYMP) and symptomatic (SYMP) patients and healthy donors. The characteristics of the SYMP, ASYMP, and healthy donor study populations used in this study, concerning age, sex, HLA-A*02:01 frequency distribution, SARS-CoV-2 positivity, and status of COVID-19 disease, are presented in Table 1. These SARS-CoV-2 positive individuals were divided into two groups: (1) HLA-A*02:01-positive SARS-CoV-2-infected ASYMP individuals, with no detectable levels of any clinical COVID-19 disease and (2) HLA-A*02:01-positive SARS-CoV-2-infected SYMP individuals with a well-documented COVID-19 clinical disease.

We analyzed the NK cell population following a gating strategy as shown in Figure 1A. The NK cell population was further categorized into CD56^dim^ and CD56^bright^ cells, and their relative frequencies were evaluated in ASYMP, SYMP, and healthy donors. Analysis of NK cell phenotype showed no difference in the mature CD56^dim^ subset in SARS-CoV-2-positive ASYMP and SYMP patients and healthy donors (Figure 1B, top panel). However, SYMP patients had significantly reduced immature CD56^bright^ NK cells (Figure 1B, bottom panel; *p* = 0.02) compared to healthy donors.

CD57 expression in NK cells defines a mature phenotype, and their expression of CD57 could also be considered a marker of terminal differentiation, although not associated with senescence in this population. It is highly expressed on CD56^dim^ cells, representing mature NK cells, whereas less than 15% of CD56^bright^ NK cells, considered immature, also express CD57 (Figure 1C). There was a significant increase in the mature (CD56^dim^/CD57^+^) and immature subset (CD56^bright^/CD57^+^) (Figure 1C, top and bottom panels; *p* = 0.03 and *p* = 0.01, respectively) in SYMP patients with COVID-19 compared with healthy controls.

These data suggest that terminal differentiation, marked by CD57 expression, is increased in both the mature (CD56^dim^) and immature (CD56^bright^) NK-cell subsets in symptomatic COVID-19 patients compared with healthy controls.

### 3.2. The Activation Status, Senescence, and Exhaustion Profile Were Significantly Increased in COVID-19 SYMP Individuals Compared to Healthy Individuals Within CD4^+^ T Cells

**Figure 2:**
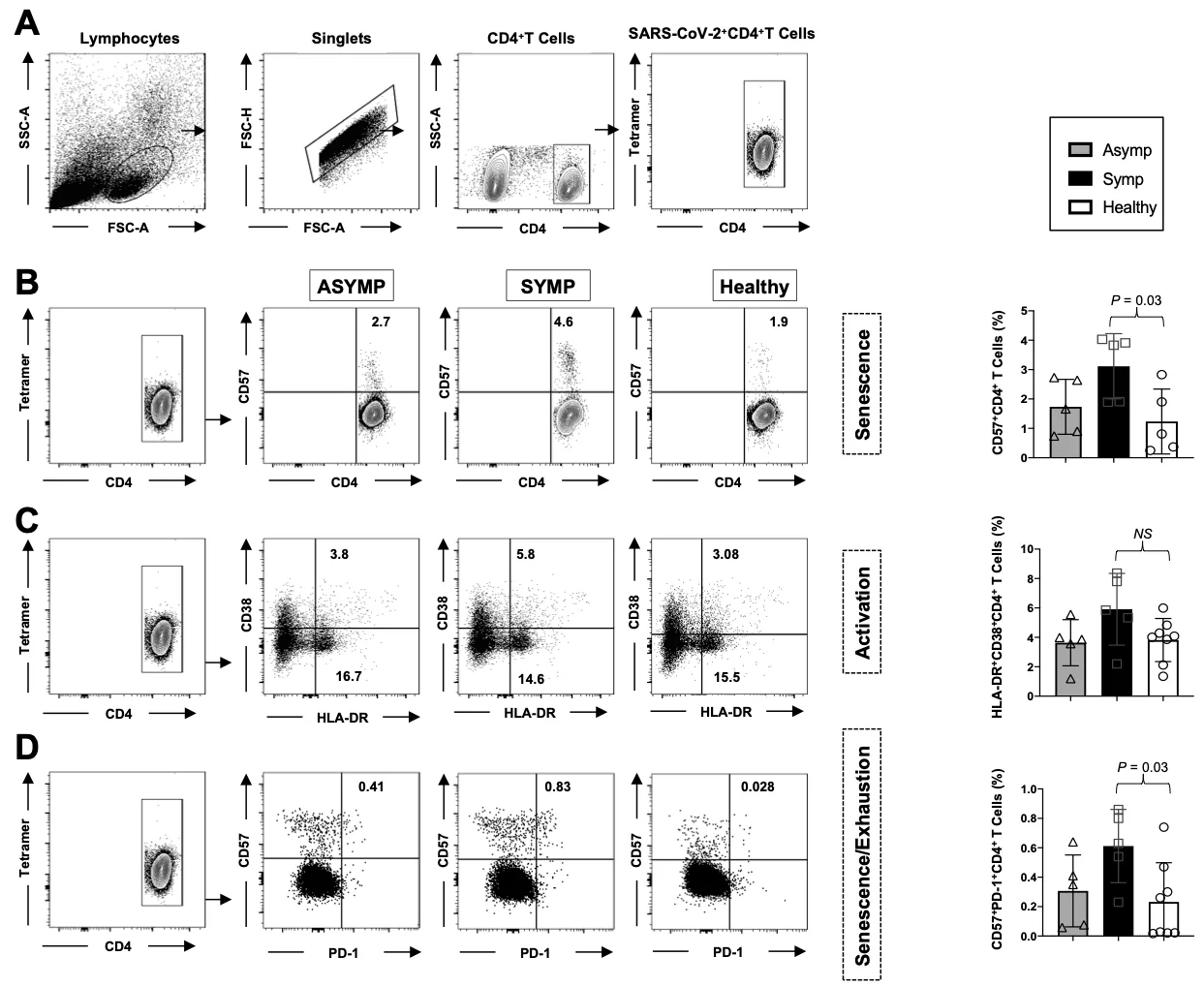
The activation status, senescence, and exhaustion profiles were significantly increased in COVID-19 SYMP individuals compared to healthy individuals within CD4^+^ T cells. Expression of CD38 and HLA-DR were detected to analyze the activation status of CD4^+^ T cells. Expression of CD57 and PD-1 were detected to analyze the senescence/exhaustion status of CD4^+^ T cells. (**A**) SARS-CoV-2-specific CD4^+^ T cells were identified using a mixture of tetramers specific to previously reported CD4^+^ T cell epitopes from nine structural and non-structural ORFs (i.e., ORF1ab, S, E, M, ORF6, ORF7a, ORF7b, ORF8, and N) [25] in the gating strategy to analyze markers related to activation status, senescence, and exhaustion together within CD4^+^ T cells. Activated cells are CD38^+^HLA-DR^+^; exhausted/senescent cells are PD1^+^CD57^+^. (**B**) Representative FACS data of the frequencies of CD57^+^ CD4^+^ T cells detected in PBMCs from COVID-19 ASYMP, SYMP, and healthy donors (**left** panel). Average frequencies of PBMC-derived CD57^+^ CD4^+^ T cells were detected in ASYMP, SYMP, and healthy donors (**right** panel). (**C**) Representative FACS data of the frequencies of HLA-DR^+^CD38^+^ CD4^+^ T cells detected in PBMCs from ASYMP individuals, SYMP individuals, and healthy donors (**left** panel). Average frequencies of PBMC-derived HLA-DR^+^CD38^+^ CD4^+^ T cells were detected in ASYMP, SYMP, and healthy donors (**right** panel). (**D**) Representative FACS data of the frequencies of CD57^+^ PD-1^+^CD4^+^ T cells detected in PBMCs from COVID-19 ASYMP, SYMP, and healthy donors (**left** panel). Average frequencies of PBMC-derived CD57^+^ PD-1^+^CD4^+^ T cells were detected from ASYMP, SYMP, and healthy donors (**right** panel). The results are representative of two independent experiments on each individual. Samples from ten ASYMP and ten SYMP patients were used, and the right columns show representative results from five ASYMP and five SYMP patients from two experiments run independently and consecutively. The indicated *p* values, calculated using an unpaired *t*-test, show statistical significance between SYMP and healthy donors.

CD4^+^ T cells in COVID-19 are activated, as characterized by the expression of cellular markers like HLA-DR and CD38. Therefore, we next evaluated the degree of CD4^+^ T cell activation in COVID-19-positive ASYMP and SYMP patients and healthy donors. Within the CD4^+^ population, we analyzed markers commonly related to T cell activation (HLA-DR and CD38). The gating strategy used to analyze markers related to activation status, senescence, and exhaustion together within CD4^+^ T cells is demonstrated in Figure 2A. The expression of CD57 correlates with senescence in human CD4^+^ and CD8^+^ T cells. Therefore, we compared the frequency of CD57^+^ on SARS-CoV-2-specific CD4^+^ T cells in COVID-19 ASYMP, SYMP, and healthy donors. PBMC-derived CD57^+^CD4^+^ T cells detected from COVID-19 SYMP individuals showed an increased frequency compared to healthy donors (Figure 2B; *p* = 0.03).

The levels of CD4^+^ T-cell activation were also evaluated in SARS-CoV-2-infected ASYMP, SYMP patients, and healthy donors. We found an increasing trend, reflected by higher proportions of HLA-DR^+^CD38^+^CD4^+^ T cells in SYMP patients compared to healthy donors; however, there was no statistical significance (Figure 2C).

Furthermore, we evaluated the expression of the senescence/exhaustion molecules on CD4^+^ T cells by analyzing markers CD57 and PD-1 (Figure 2D). The proportion of CD57^+^PD-1^+^CD4^+^ T cells was significantly higher in COVID-19-positive SYMP patients than in healthy donors (Figure 2D; *p* = 0.03). However, no statistical difference was detected in the proportion of CD57^+^PD-1^+^CD4^+^ T cells between SYMP and ASYMP COVID-19 patients.

Taken together, these findings show that CD57^+^PD-1^+^ CD4^+^ T cells, reflecting an exhausted/senescent phenotype, are significantly increased in COVID-19-positive SYMP patients compared with healthy donors, while CD4^+^ T cell activation showed only a non-significant increasing trend in this cohort.

### 3.3. Frequent SARS-CoV-2-Specific Senescent CD4^+^ T Cells with an Effector Memory Phenotype (CD57^+^CD4^+^ T_EM_ and CD57^+^CD4^+^ T_EMRA_ Cells) Were Detected in COVID-19 SYMP Individuals Compared to Healthy Individuals

**Figure 3:**
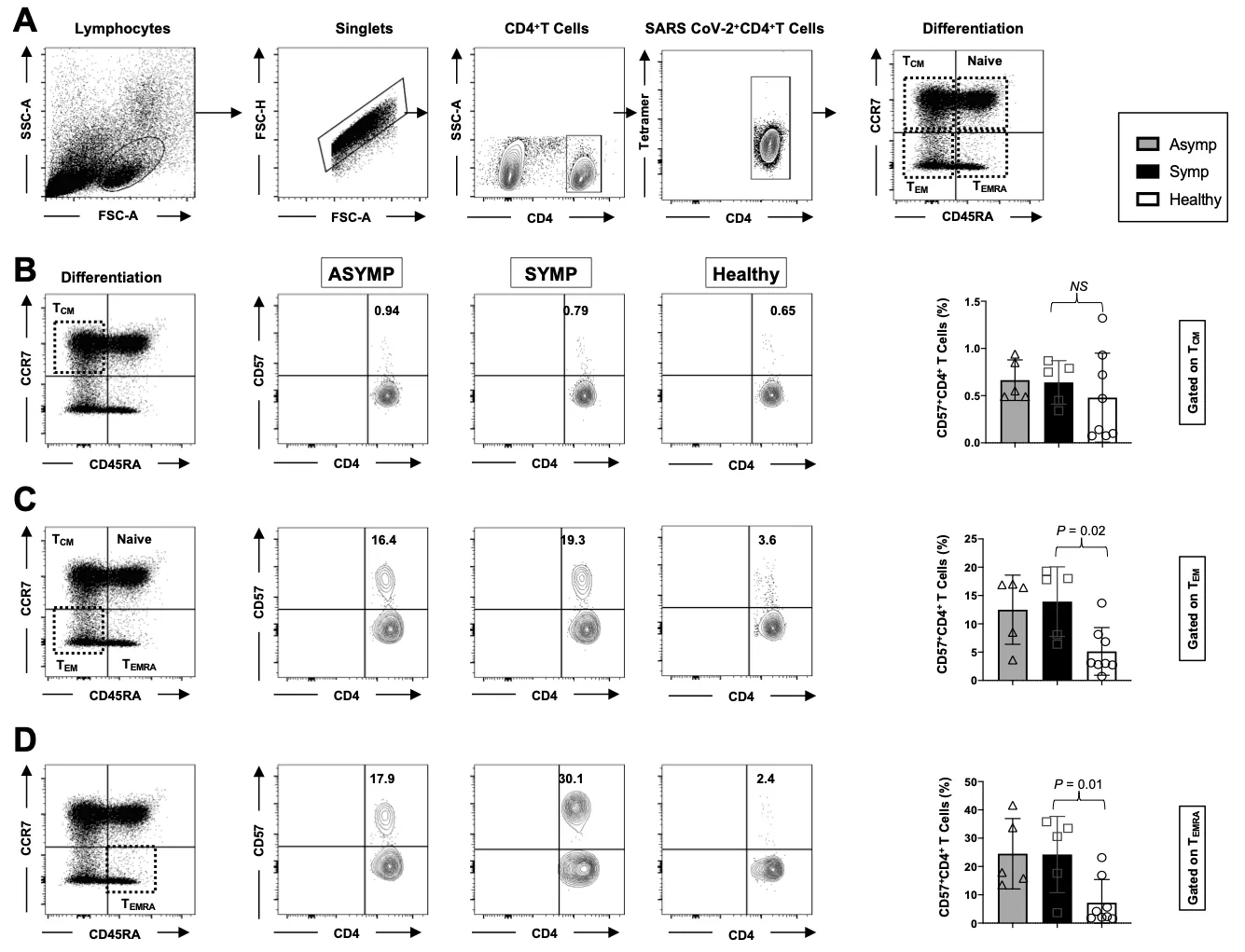
Frequent SARS-CoV-2-specific senescent CD4^+^ T cells with an effector memory phenotype (CD57^+^CD4^+^ T_EM_ and CD57^+^CD4^+^ T_EMRA_ cells) detected in COVID-19 SYMP individuals compared to healthy individuals. The phenotype of SARS-CoV-2-specific CD4^+^ T cells, using a mixture of tetramers specific to previously reported CD4^+^ T cell epitopes identified in nine structural and non-structural ORFs (i.e., ORF1ab, S, E, M, ORF6, ORF7a, ORF7b, ORF8, and N) [25] (**A**) Gating strategy showing analysis in T_NAIVE_, T_CM_, T_EMRA_, and T_EM_ phenotypes in PBMCs from COVID-19 ASYMP, SYMP, and healthy donors. Representative FACS data (**left** panel) and the frequencies of CD57 (**right** panel) (**B**) gated on CD45RA^low^CCR7^high^CD4^+^ T_CM_ cells, (**C**) gated on CD45RA^low^CCR7^low^CD4^+^ T_EM_ cells, and (**D**) and CD45RA^high^CCR7^low^CD4^+^ T_EMRA_ cells detected in COVID-19 ASYMP individuals, SYMP individuals, and healthy donors. The results are representative of two independent experiments on each individual. Samples from ten ASYMP and ten SYMP patients were used, and the right columns show representative results from five ASYMP and five SYMP patients from two experiments run independently and consecutively. The indicated *p* values, calculated using an unpaired *t*-test, show statistical significance between SYMP and healthy donors.

SARS-CoV-2-specific memory CD4^+^ T cells were also categorized into three major phenotypically distinct effector memory (T_EM_), central memory (T_CM_), and a subset of effector memory T cells that re-express CD45RA subpopulations termed T_EMRA_. Moreover, we studied the expression levels of CD57 on the memory CD4^+^ T cell subpopulations at various stages of differentiation: central memory T cells (CD45RA^low^CCR7^high^CD4^+^ T_CM_ cells); effector memory T cells (CD45RA^low^CCR7^low^CD4^+^ T_EM_ cells); and T_EMRA_ T cells (CD45RA^high^CCR7^low^CD4^+^ T_EMRA_ cells). In the peripheral blood of HLA-A*02:01-positive, SARS-CoV-2-positive ASYMP, SYMP, and healthy donors, we compared the CD57 expression in CD4^+^ T cells and divided them into T_NAIVE_, T_CM_, T_EM,_ and T_EMRA_ phenotypes (Figure 3A). Similar percentages of CD57^+^CD45RA^low^CCR7^high^CD4^+^ T_CM_ cells were detected in ASYMP, SYMP, and healthy donors (Figure 3B; left and right panels). There was an increase in the CD57^+^CD45RA^low^CCR7^low^CD4^+^ T_EM_ cells in SYMP individuals compared to healthy controls (Figure 3C; left and right panels, *p* = 0.02). Significantly higher percentages of CD57^+^CD45RA^high^CCR7^low^CD4^+^ T_EMRA_ cells (Figure 3D; left and right panels) were detected in SYMP individuals compared to healthy donors (*p* = 0.01). Altogether, the phenotypic properties of memory CD4^+^ T cells revealed increased frequencies of CD57-expressing effector memory (T_EM_) and T_EMRA_ subsets, but not central memory (T_CM_) cells, in SYMP compared with healthy donors. SYMP individuals appeared to develop frequencies of effector memory CD57^+^CD4^+^ T_EMRA_ and CD57^+^CD4^+^ T_EM_ cells similar to ASYMP individuals, with both groups elevated compared to healthy donors.

### 3.4. Frequent SARS-COV-2 S_1220–1228_ and S_958–966_ Epitope-Specific CD57^+^CD8^+^ T Cells Detected in COVID-19 SYMP Individuals Compared to ASYMP and Healthy Individuals

**Figure 4:**
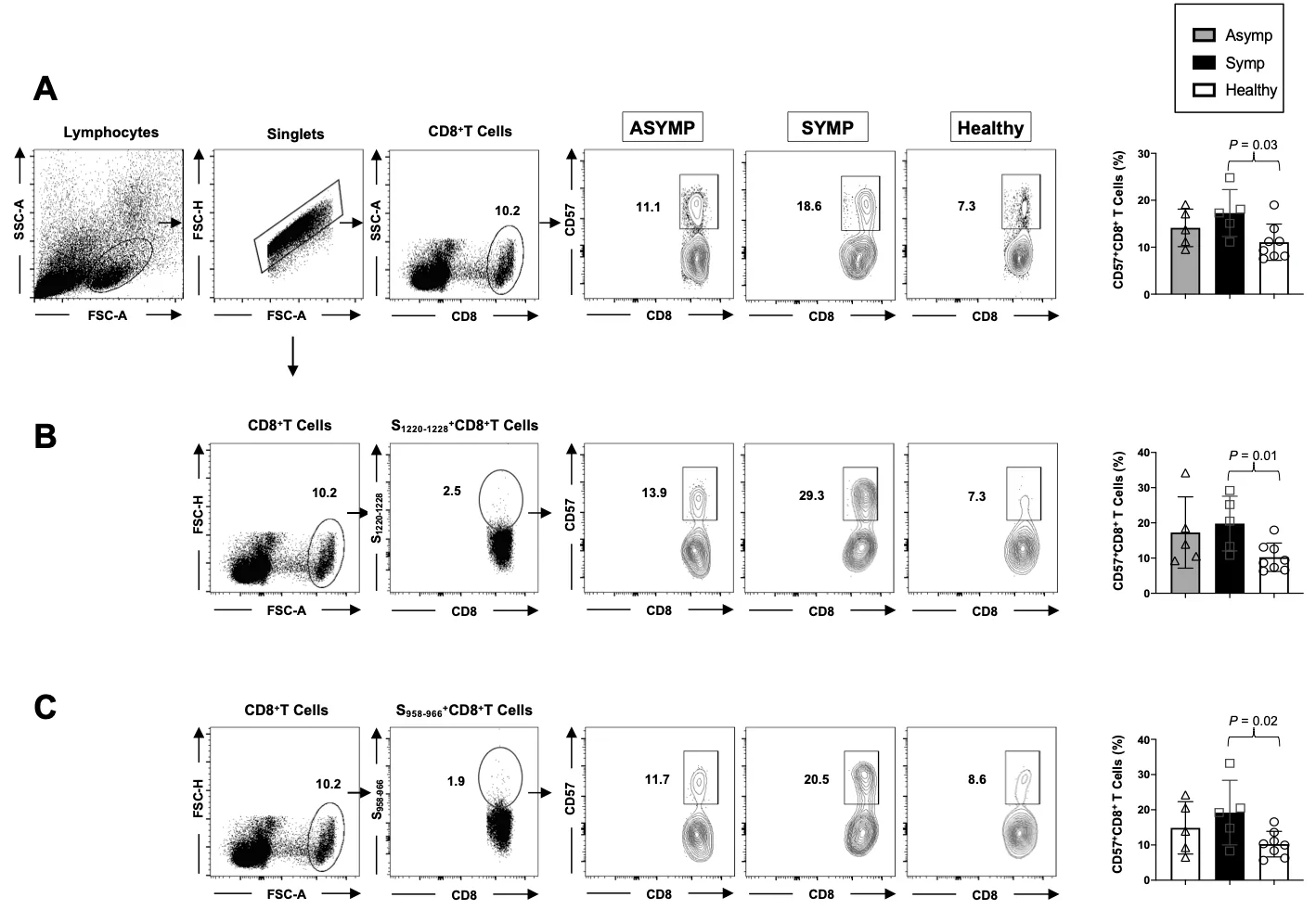
Frequent SARS-COV-2 S_1220–1228_ and S_958–966_ epitope-specific CD57^+^CD8^+^ T cells detected in COVID-19 SYMP individuals compared to ASYMP and healthy individuals. The frequency of CD57^+^ on total CD8^+^ T cells and SARS-CoV-2 peptide/tetramer complex specific CD8^+^ T cells were analyzed in HLA-A*02:01-positive COVID-19 ASYMP, SYMP, and healthy donors. (**A**) Gating strategy used to analyze markers related to senescence gated within CD8^+^ T cells from COVID-19 ASYMP, SYMP, and healthy individuals (**left** panel), and average frequencies of PBMC-derived CD8^+^ T cells detected from COVID-19 ASYMP, SYMP and healthy donors (**right** panel). (**B**) Representative FACS data of the frequencies of CD57^+^CD8^+^ T cells, specific to the S_1220–1228_ epitope, detected in PBMCs from HLA-A*02:01-positive COVID-19 ASYMP, SYMP, and healthy individuals (**left** panel). Average frequencies of PBMC-derived CD8^+^ T cells specific to the S_1220–1228_ epitope were detected from COVID-19 ASYMP, SYMP, and healthy donors (**right** panel). (**C**) Representative FACS data of the frequencies of CD57^+^CD8^+^ T cells, specific to S_958–966_ epitope, detected in PBMCs from HLA-A*02:01 positive COVID-19 ASYMP, SYMP, and healthy donors (**left** panel). Average frequencies of PBMC-derived CD57^+^CD8^+^ T cells, specific to the S_958–966_ epitope, were detected from ASYMP, SYMP, and healthy donors (**right** panel). The results are representative of two independent experiments on each individual. Samples from ten ASYMP and ten SYMP patients were used, and the right columns show representative results from five ASYMP and five SYMP patients from two experiments run independently and consecutively. The indicated *p* values, calculated using an unpaired *t*-test, show statistical significance between SYMP and healthy donors.

As described earlier, SARS-CoV-2-positive individuals were segregated into two groups: (1) HLA-A*02:01-positive SARS-CoV-2-infected ASYMP individuals, and (2) HLA-A*02:01-positive SARS-CoV-2-infected SYMP individuals with a well-documented COVID-19 clinical disease. We next compared the frequency of CD57^+^ on total CD8^+^ T cells in HLA-A*02:01-positive COVID-19 ASYMP, SYMP, and healthy donors. We have used a gating strategy to analyze markers related to senescence (CD57^+^) gated within CD8^+^ T cells from COVID-19 ASYMP, SYMP, and healthy donors. Average frequencies of PBMC-derived CD57^+^CD8^+^ T cells detected from COVID-19 SYMP individuals showed an increased frequency compared to healthy donors (Figure 4A, *p* = 0.03). We then compared the frequency of SARS-CoV-2 peptide/tetramer complex-specific CD8^+^ T cells. The representative dot plots in Figure 4B indicate an increased frequency of CD57^+^CD8^+^ T cells, specific to the S1220–1228 epitope in COVID-19 SYMP individuals compared to ASYMP healthy donors (*p* = 0.01). Similarly, Figure 4C depicts the high frequencies of CD57^+^CD8^+^ T cells detected in COVID-19 SYMP individuals against another peptide/tetramer complex S_958–966_ epitope (*p* = 0.02). Altogether, these results indicate that SYMP individuals develop frequent SARS-CoV-2-specific CD57^+^CD8^+^ T cells compared to ASYMP and healthy donors.

### 3.5. Frequent SARS-CoV-2 S_1220–1228_ Epitope-Specific Senescent CD8^+^ T Cells with an Effector Memory Phenotype (CD57^+^CD8^+^ T_EM_ and CD57^+^CD8^+^ T_EMRA_ Cells) Detected in COVID-19 SYMP Individuals Compared to Healthy Individuals

**Figure 5:**
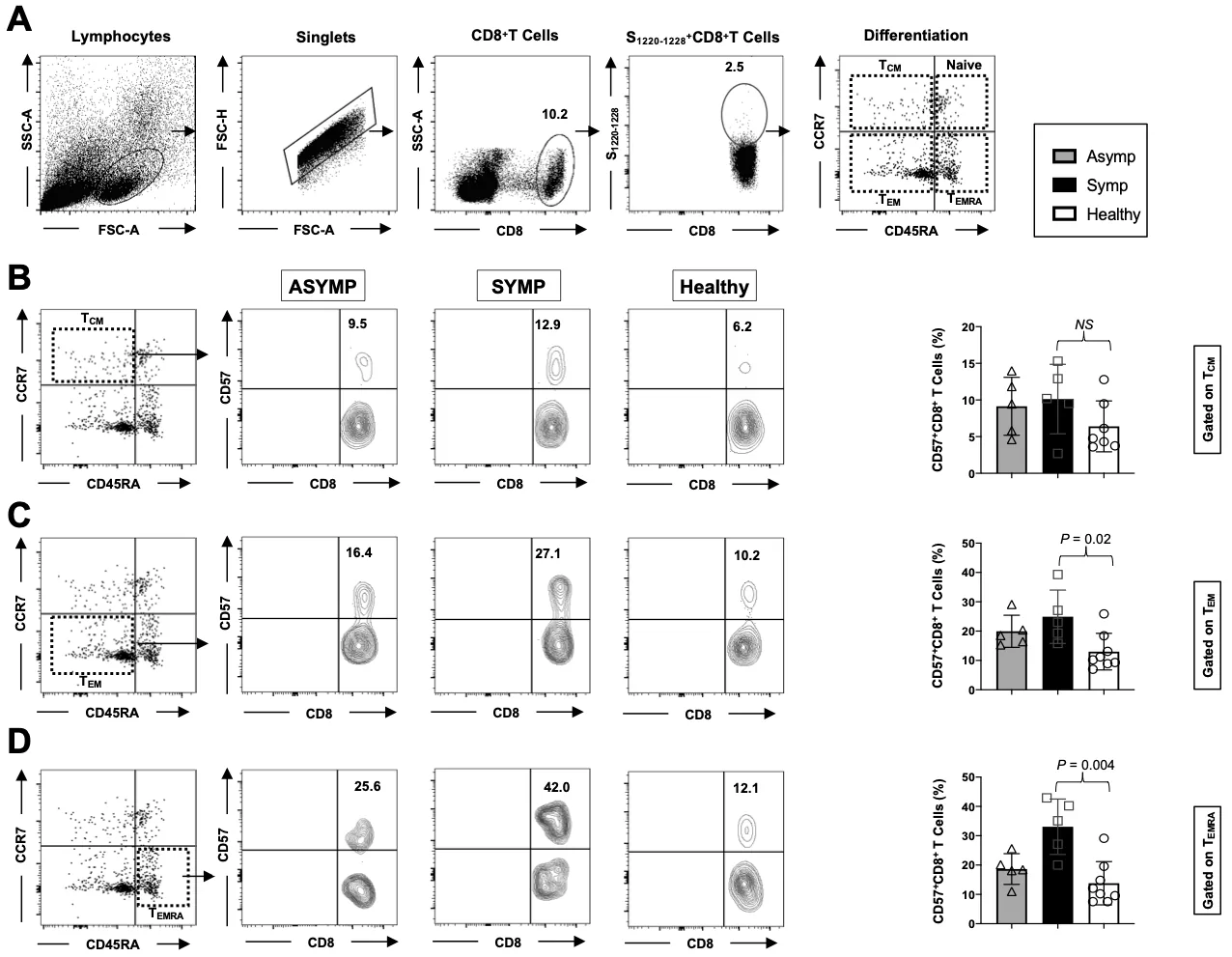
Frequent SARS-CoV-2 S_1220–1228_ epitope-specific senescent CD8^+^ T cells with an effector memory phenotype (CD57^+^CD8^+^ T_EM_ and CD57^+^CD8^+^ T_EMRA_ cells) detected in COVID-19 SYMP individuals compared to healthy individuals. The phenotype of CD8^+^ T cells specific to S_1220–228_ peptide/tetramer shown in (**A**) was analyzed in terms of T_NAIVE_, T_CM_, T_EMRA,_ and T_EM_ phenotypes in PBMCs from HLA-A*02:01-positive COVID-19 ASYMP, SYMP, and healthy donors. Representative FACS data (**left** panel) and the frequencies of CD57 (**right** panel) gated on CD45RA^low^CCR7^high^CD8^+^ T_CM_ cells (**B**), gated on CD45RA^low^CCR7^low^CD8^+^ T_EM_ cells (**C**), and CD45RA^high^CCR7^low^CD8^+^ T_EMRA_ cells (**D**) detected in COVID-19 ASYMP, SYMP, and healthy donors. The results are representative of two independent experiments on each individual. Samples from ten ASYMP and ten SYMP patients were used, and the right columns show representative results from five ASYMP and five SYMP patients from two experiments run independently and consecutively. The indicated *p* values, calculated using an unpaired *t*-test, show statistical significance between SYMP and healthy donors.

Similar to the CD4^+^ T cell memory response, SARS-CoV-2-specific memory CD8^+^ T cells are also categorized into three major phenotypically distinct subpopulations termed TEMRA: effector memory (TEM), central memory (TCM), and a subset of effector memory T cells that re-express CD45RA. Furthermore, we examined the expression levels of CD57 on the memory CD8^+^ T cell subpopulations at various stages of differentiation: central memory T cells (CD45RA^low^CCR7^high^CD8^+^ T_CM_ cells); effector memory T cells (CD45RA^low^CCR7^low^CD8^+^ T_EM_ cells); and TEMRA T cells (CD45RA^high^CCR7^low^CD8^+^ T_EMRA_ cells). In the peripheral blood of HLA-A*02:01-positive, SARS-CoV-2-positive ASYMP, SYMP, and healthy donors, we compared the CD57 expression in CD8^+^ T cells specific to the S_1220–1228_ epitope and divided them into T_NAIVE_, T_CM_, T_EM,_ and T_EMRA_ phenotypes (Figure 5A). Similar percentages of CD57^+^CD45RA^low^CCR7^high^CD8^+^ T_CM_ cells were detected in ASYMP, SYMP and healthy donors (Figure 5B; left and right panel). There was a significant increase in the CD57^+^CD45RA^low^CCR7^low^CD8^+^ T_EM_ cells in SYMP individuals compared to healthy donors (Figure 5C; left and right panels). Significantly higher percentages of CD57^+^CD45RA^high^CCR7^low^CD8^+^ T_EMRA_ cells (Figure 5D; left and right panels) were detected in SYMP individuals compared to healthy donors (*p* = 0.004). Altogether, the phenotypic properties of SARS-CoV-2 S_1220–1228_ epitope-specific memory CD8^+^ T cells revealed increased frequencies of CD57^+^ effector memory (T_EM_) and T_EMRA_ subsets, but not central memory (T_CM_) cells, in SYMP compared with healthy donors. These data suggest that SYMP individuals accumulate terminally differentiated, SARS-CoV-2-specific effector memory CD8^+^ T cells, a phenotype associated with reduced proliferative and cytotoxic capacity, distinguishing symptomatic from healthy immune responses to SARS-CoV-2.

### 3.6. The Activation Status, Senescence, and Exhaustion Profile Were Significantly Increased in COVID-19 SYMP Individuals Compared to Healthy Individuals Within CD8^+^ T Cells

**Figure 6:**
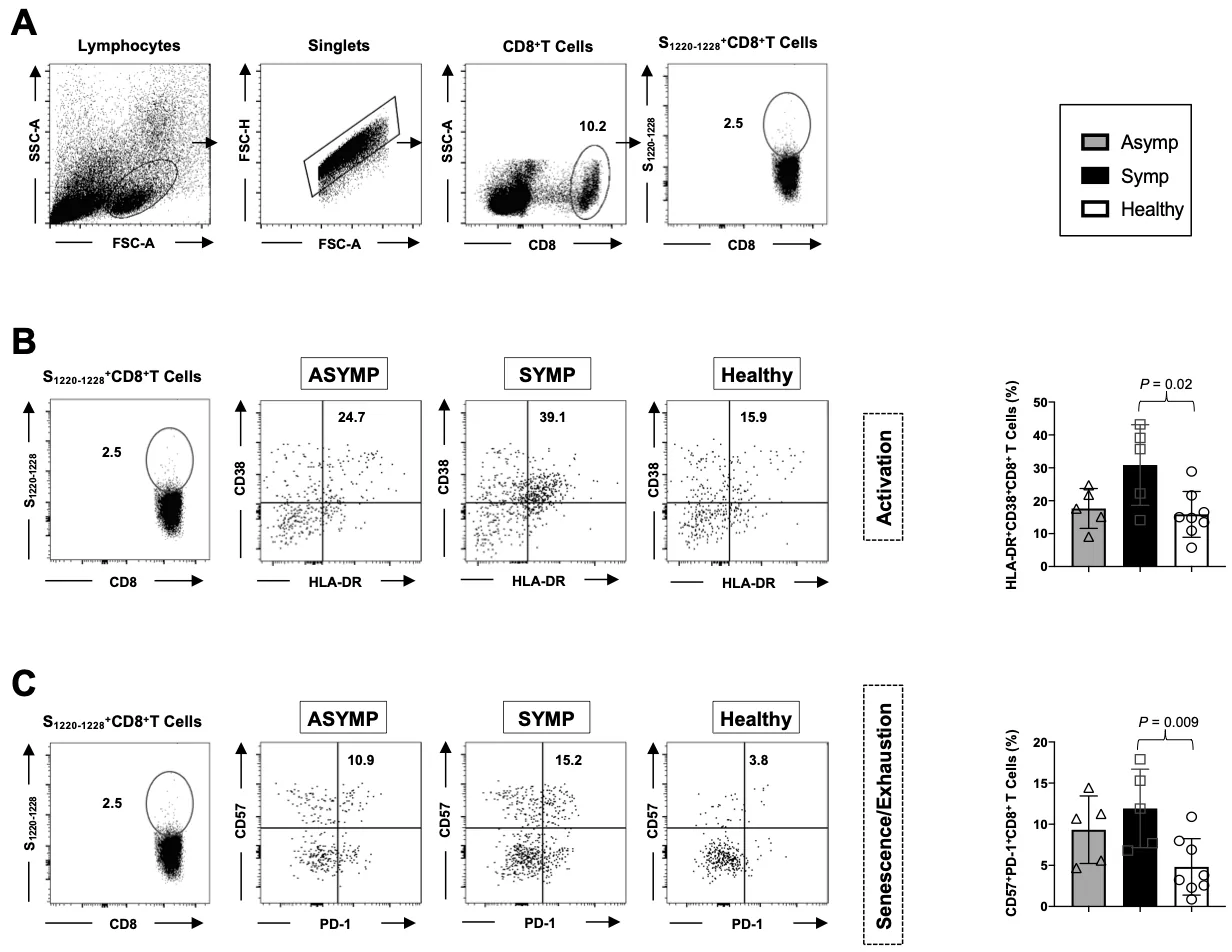
The activation status, senescence, and exhaustion profile were significantly increased in COVID-19 SYMP individuals compared to healthy individuals within CD8^+^ T cells. Expression of CD38 and HLA-DR was detected to analyze the activation status of CD8^+^ T cells. Expression of CD57 and PD-1 was detected to analyze the senescence/exhaustion status of CD8^+^ T cells. (**A**) Gating strategy used to analyze markers related to activation status, senescence, and exhaustion together within SARS-CoV-2-specific CD8^+^ T cells. Activated cells are CD38^+^HLA-DR^+^; exhausted/senescent cells are PD1^+^CD57^+^. FACS was used to determine the expression level of various markers on tetramer-gated CD8^+^ T cells specific to the S_1220–1228_ epitope. (**B**) Representative FACS data of the frequencies of HLA-DR^+^CD38^+^ CD8^+^ T cells, specific to the S_1220–1228_ epitope, detected in PBMCs from HLA-A*02:01 positive COVID-19 ASYMP individuals, SYMP individuals, and healthy donors (**left** panel). Average frequencies of PBMC-derived HLA-DR^+^CD38^+^ CD8^+^ T cells, specific to the S_1220–1228_ epitope, were detected in COVID-19 ASYMP, SYMP, and healthy donors (**right** panel). (**C**) Representative FACS data of the frequencies of CD57^+^ PD-1^+^CD8^+^ T cells, specific to the S_1220–1228_ epitope, detected in PBMCs from HLA-A*02:01-positive COVID-19 ASYMP individuals, SYMP individuals, and healthy donors (**left** panel). Average frequencies of PBMCs-derived CD57^+^ PD-1^+^CD8^+^ T cells, specific to the S_1220–1228_ epitope, were detected from ASYMP, SYMP, and healthy donors (**right** panel). The results are representative of two independent experiments on each individual. Samples from ten ASYMP and ten SYMP patients were used, and the right columns show representative results from five ASYMP and five SYMP patients from two experiments run independently and consecutively. The indicated *p* values, calculated using an unpaired *t*-test, show statistical significance between SYMP and healthy donors.

Most viral infections induce activation of CD8^+^ T cells that can be detected by increases in the co-expression of CD38 and Human leukocyte antigen-DR isotype (HLA-DR). HLA-DR is constitutively expressed by antigen-presenting cells (APCs) and is involved in the presentation of antigens to T-cells. Most T-cells do not express it, but notably, a subset of activated T-cells becomes HLA-DR^+^ during an immune response. In contrast, CD38 is constitutively expressed by naive T-cells, down-regulated in resting memory cells, and then elevated again in activated cells. Thus, we evaluated the degree of CD8^+^ T-cell activation in COVID-19-positive ASYMP and SYMP patients and healthy donors. Within the CD8^+^ population, we analyzed markers commonly related to T cell activation (HLA-DR and CD38). Our gating strategy was used to analyze markers related to activation status, senescence, and exhaustion together within SARS-CoV-2-specific CD8^+^ T cells (Figure 6A). The levels of T-cell activation were significantly higher (hyperactivated) in SARS-CoV-2-infected SYMP patients than in healthy donors, as reflected by the higher proportions of HLA-DR^+^CD38^+^CD8^+^ T cells (Figure 6B, *p* = 0.02).

We evaluated the senescence/exhaustion molecules’ expression in circulating T cells by analyzing the markers CD57 and PD-1 (Figure 6C). We found that the proportion of CD57^+^PD-1^+^CD8^+^ T cells was significantly higher in COVID-19 SYMP patients compared to healthy donors (Figure 6C, *p* = 0.009). However, there was no statistical difference in the proportion of CD57^+^PD-1^+^CD8^+^ T cells between ASYMP and SYMP patients (Figure 6C).

Taken together, these findings show that increased CD8^+^ T cell activation (HLA-DR^+^CD38^+^) and terminal differentiation/exhaustion (CD57^+^PD-1^+^) are prominent features of COVID-19-positive SYMP individuals.

### 3.7. Elevated Plasma Levels of Selected Cytokines in COVID-19 ASYMP and SYMP Individuals Compared to Healthy Controls

**Figure 7:**
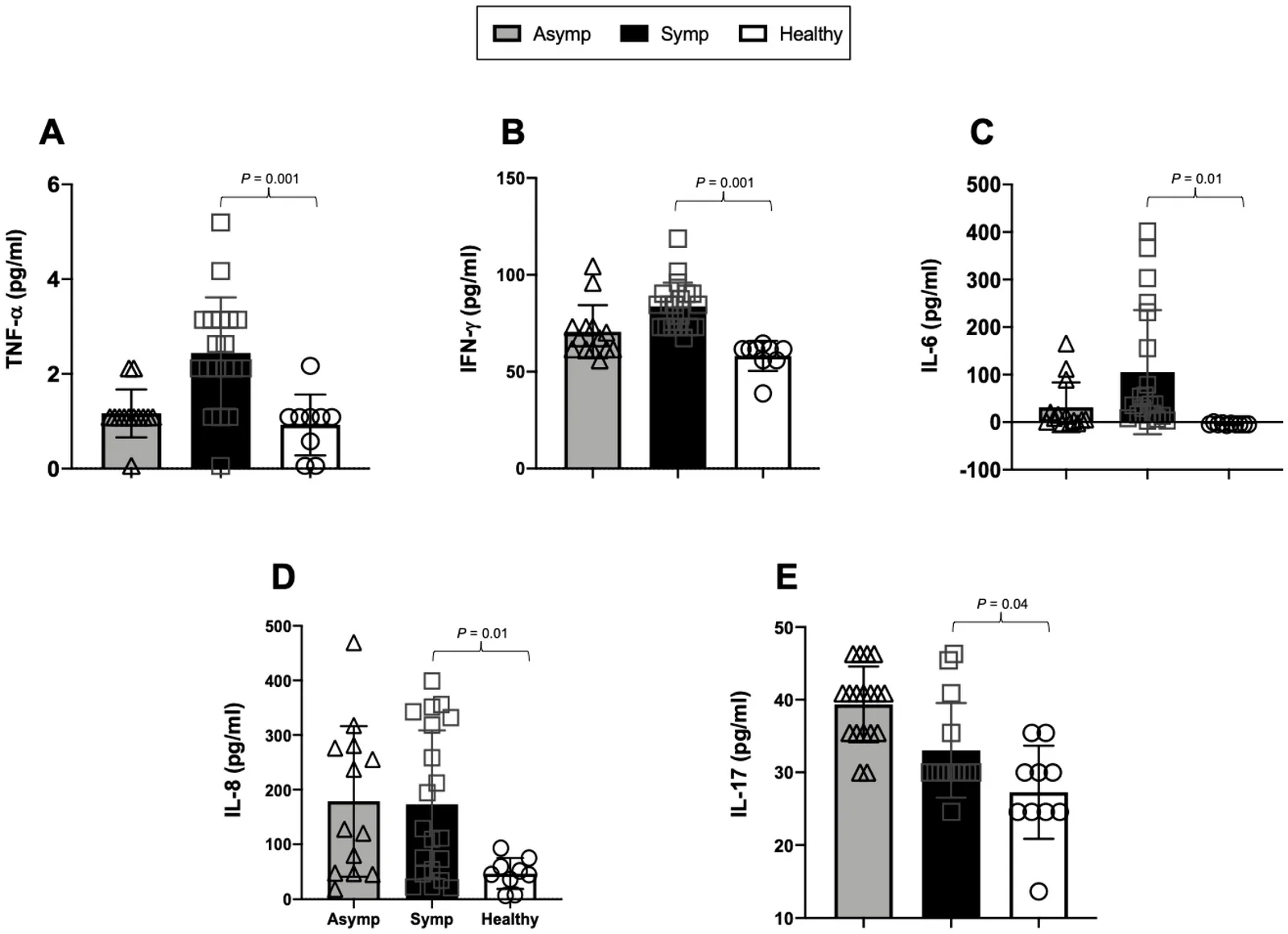
Elevated plasma levels of selected cytokines in COVID-19 ASYMP and SYMP individuals compared to healthy controls. Cytokine expression levels of two replicates per sample were measured in plasma samples of COVID-19 ASYMP, SYMP, and healthy donors using Luminex. (**A**) Bar graphs with individual values showing the average amount of TNF-α (pg/mL) produced by ASYMP, SYMP, and healthy donors. (**B**) Bar graphs with individual values showing the average amount of IFN-γ(pg/mL) produced by ASYMP, SYMP, and healthy donors. (**C**) Bar graphs with individual values show the average IL-6 (pg/mL) produced by ASYMP, SYMP, and healthy donors. (**D**) Bar graphs with individual values show the average IL-8 (pg/mL) produced by ASYMP, SYMP, and healthy donors. (**E**) Bar graphs with individual values show the average IL-17 (pg/mL) produced by ASYMP, SYMP, and healthy donors. Shown are the results from ten ASYMP and ten SYMP patients in two consecutive experiments. The indicated *p* values, calculated using an unpaired *t*-test, show statistical significance between SYMP and healthy donors.

Many studies have previously reported that the hyper-inflammatory response induced by SARS-CoV-2 is a major cause of disease severity and death. Therefore, we implemented a multiplex cytokine assay (Luminex) to measure inflammatory cytokines known to contribute to pathogenic inflammation (IL)-6, IL-8, tumor necrosis factor (TNF)-α, interferon (IFN)-γ and IL-17. In the plasma samples of COVID-19 ASYMP, SYMP, and healthy donors, the cytokines assessed in this study had different detection ranges, with IL-6 and IL-8 having the most dynamic profile, followed by TNF-α, IL-17, and IFN-γ. We found that TNF-α and IFN-γ (*p* = 0.001) were significantly elevated in COVID-19 symptomatic patients compared to healthy donors (Figure 7A,B). Similarly, we found that IL-6 and IL-8 (*p* = 0.01) were significantly elevated in COVID-19 symptomatic patients compared to healthy donors (Figure 7C,D). Interleukin (IL)-17 is one of the many cytokines released during SARS-CoV-2 infection. IL-17 plays a crucial role in neutrophil recruitment and activation. Neutrophils can subsequently migrate to the lung and are heavily involved in the pathogenesis of COVID-19 [29]. We found that SARS-CoV-2-positive ASYMP and SYMP individuals had significantly higher levels of IL-17 than healthy donors (*p* = 0.04) (Figure 7E).

The vast majority of SYMP patients demonstrated elevated levels of selected proinflammatory cytokines (TNF-α, IFN-γ, IL-6, IL-8, and IL-17) compared with healthy donors. In contrast, cytokine levels were not significantly different between COVID-19 ASYMP and SYMP individuals.

Overall, our findings report that NK, CD4^+^ T cells, and CD8^+^ T cells’ phenotypic and functional characteristics in severe COVID-19 infection were compatible with the activation of dysfunction/exhaustion pathways.

## 4. Discussion

The immunological hallmark of severe COVID-19 is not simply the loss of lymphocytes, but the functional collapse of those that remain. While COVID-19 drives profound lymphopenia and broad alterations in T cell phenotypes, the quality of antigen-specific T cell responses, not their quantity alone, has emerged as the critical determinant of viral clearance, disease trajectory, and the capacity to generate durable immunological memory protective against reinfection. These insights carry direct implications for vaccine design, where eliciting functionally competent, long-lived T cell responses remains a central and unresolved challenge [30]. Beyond the acute phase, post-infection remodeling of T cell subset composition and function carries lasting consequences for patients’ long-term immune competence, consequences that are only beginning to be appreciated [31,32,33,34].

Central to this dysfunction is the progressive acquisition of inhibitory and senescent markers that erode T cell effector capacity precisely when it is most needed. Among these, CD57 has emerged as a key marker of replicative senescence on T cells and NK cells, identifying terminally differentiated populations characterized by loss of the costimulatory receptor CD28, diminished proliferative capacity, and a paradoxical inability to eradicate infection despite retaining cytotoxic machinery [21,35,36]. This senescent transition is not incidental: repeated antigen stimulation drives a stepwise loss of CD28, essential for T cell activation via B7 co-stimulatory signaling, and the corresponding upregulation of CD57, marking cells that are functionally exhausted and replicationally inert [37,38,39]. Understanding how this exhaustion–senescence axis shapes the immune response to SARS-CoV-2 across the full spectrum of clinical severity is therefore essential, both for defining the immunopathogenesis of severe COVID-19 and for identifying the therapeutic leverage points needed to restore protective immunity [40,41,42,43].

The CD57 antigen is commonly used to identify populations of late-differentiated ‘senescent’ cells with defined cell phenotypes and effector functions [44,45]. In this report, we examined the patterns of expression of CD57 on NK cells and SARS-CoV-2-specific T-cells and determined increased expression of these exhausted and senescent markers in symptomatic individuals compared to those with asymptomatic infections and healthy donors. While CD57 is now well-recognized as a marker for terminally differentiated T-cells, it was originally thought to identify cells with natural killer activity. The expression of CD57 varies among NK cell subsets. NK cells are innate effector lymphocytes that respond to acute viral infections but might also contribute to immunopathology. NK cells are typically divided into CD56^bright^ NK cells and CD56^dim^ NK cells, which rapidly respond during diverse acute viral infections in humans, including against dengue virus, hantavirus, tick-borne encephalitis virus, and yellow fever virus, among others [46,47,48]. Although a similar analysis of NK cells has not been performed in acute SARS-CoV-2 infection-causing COVID-19, early reports from the pandemic (in line with our findings) have indicated low circulating NK cell numbers in patients with moderate and severe disease [49,50,51]. The SARS-CoV-2 infection has also been linked to reduced NK cell counts during the acute phase of infection. We determined a terminally differentiated phenotype with upregulated levels of CD57 molecules in NK cells from SYMP COVID-19 patients. CD57 is expressed by CD16^pos^CD56^dim^ cytotoxic NK cells and CD16^pos^CD56^neg^ inflammatory NK cells, whereas CD16^neg^CD56^bright^ regulatory NK cells do not express this marker even during chronic infections [52,53,54]. The acquisition of CD57 thus follows the natural differentiation of NK cells (from regulatory to cytotoxic to inflammatory NK cells). Thus, like T cells, NK cell expression of CD57 could be considered a marker of terminal differentiation, albeit not associated with senescence in this population.

The majority of prior studies into the biology of CD57 focused on the antigen’s significance in distinct T cell subsets. In the late phases of differentiation, CD57 has been found on both CD4 and CD8 T cells [21]. CD57 identifies terminally differentiated cells with decreased proliferative responses in CD8 T lymphocytes. T cell senescent markers were more associated with CD8^+^ T cells than CD4^+^ T cells, consistent with our results that accumulate at lower frequencies for CD4^+^ T cells in the human periphery [55]. Our findings herein indicate an increased expression of CD57^+^ T cell subsets in symptomatic patients. It was previously shown that PD-1^+^CD57^+^CD8^+^ T cells had increased sensitivity to apoptosis mediated by PD-1 [56].

The increased expression of CD57 and PD-1 double-positive markers on CD8^+^ T cells in COVID-19 suggests that these cells are at a higher risk of apoptosis. The fraction of T cells that express CD57 increased in symptomatic individuals, suggesting that the observed phenotypic changes may lower the T cell repertoire’s responsiveness to SARS-CoV-2 antigens, resulting in an impaired ability to eliminate the infection. CD57^+^ memory T cells accumulate in peripheral blood throughout life, especially after infection with CMV [57]. These associations with age and persistent antigenic drive were mechanistically linked in an in vitro study, which reported that replicative senescent memory CD8^+^ T cells expressed CD57 [21]. However, an earlier study had reached a different conclusion [58], and later experiments showed that CD57^+^ memory CD8^+^ T cells could proliferate in vitro in the presence of certain growth factors, potentially mimicking the in vivo microenvironment [59]. Previous studies suggest that T_EMRA_ cells are fairly resistant to apoptosis and remain in the CD8^+^ lineage for an estimated half-life of about 25 years, assuming a simple exponential decline without phenotypic change [60,61]. CD8^+^ T_EMRA_ cells that expressed CD57 were reported to be more sensitive to cell death than CD8^+^ T_EMRA_ cells that lacked CD57 in response to severe stimulation with supraphysiological doses of phytohemagglutinin and interleukin-2 [17,62]. Studies showed equivalent frequencies of antigen-specific T cells in asymptomatic and symptomatic individuals, but asymptomatic individuals showed proportionate secretion of IFN-γ, IL-10, and pro-inflammatory cytokines (IL-6, TNF-α, IL-1β), whereas their secretion was disproportionate in symptomatic individuals [6,7,8]. Compared to asymptomatic individuals and healthy donors, symptomatic patients had increased CD57 expression in CD8^+^ T_EMRA+_ memory cells. Compared to asymptomatic infections, the phenotypic abnormality of T cells during COVID-19 infections is more apparent in symptomatic individuals and is associated with higher expression levels of exhausted and senescent markers.

We report that the CD57^+^CD4^+^ T_EM_ and T_EMRA_ populations were similarly elevated in ASYMP and SYMP individuals relative to healthy donors, rather than distinguishing the two infected groups from one another. Rather than diminishing its value, the inclusion of the ASYMP cohort here serves an important control function: because ASYMP individuals were never hospitalized and did not experience the physiological stress, inflammation, or clinical interventions associated with critical illness, the presence of comparable CD57^+^ TEM/TEMRA frequencies in this group indicates that terminal differentiation within the CD4^+^ memory T cell compartment is driven by SARS-CoV-2 infection and antigen exposure itself, rather than being a secondary consequence of severe disease or its treatment. This contrasts with phenotypes reported elsewhere in this study that more specifically track with symptomatic critical illness, and highlights that not all senescence-associated changes are severity-specific. The findings of this research contribute to the current knowledge of the innate and adaptive immune landscape in asymptomatic and symptomatic COVID-19 patients. However, we recognize limitations that could be addressed with bigger sample sizes and matched control groups. Furthermore, the phenotype and activity of immune cells from the lungs performing a direct role in establishing symptomatic infections are unknown. As a result, the immunophenotypic traits in the lungs may not completely mirror the hierarchy of immunodominant circulating immune cells in the blood.

This study evaluated a relatively large number of immune parameters across the healthy donors, ASYMP, and SYMP groups using unadjusted Student’s *t*-tests and one-way ANOVA, without correction for multiple hypothesis testing. We therefore consider this first report exploratory and hypothesis-generating, with the immune phenotypes identified here warranting validation in larger, independent cohorts using pre-specified, multiplicity-adjusted statistical analyses. Because this study used a cross-sectional design with PBMCs sampled at a single time point, it could not establish whether the NK and CD8^+^ T cell exhaustion/senescence phenotypes we describe are a cause or a consequence of severe COVID-19. On one hand, pre-existing differences in immune composition, such as those associated with age or prior antigen experience, are known to shape the magnitude and quality of the SARS-CoV-2-specific T cell response and to correlate independently with disease severity, raising the possibility that some individuals enter infection with an immune landscape already predisposed toward the phenotypes we observed [63]. On the other hand, longitudinal profiling of hospitalized COVID-19 patients has shown that lymphocyte dysfunction, including impaired proliferative and cytotoxic capacity, evolves dynamically over the course of severe disease, arguing that at least part of this signature emerges as a downstream consequence of high viral burden and sustained inflammation rather than preceding it [64]. Our cross-sectional data cannot distinguish between these possibilities, and the CD57^+^ exhaustion/senescence signatures reported here should therefore be interpreted as correlates rather than established causes of disease severity. Longitudinal sampling from the time of infection through to recovery will be required to resolve this question.

A limitation is that this study restricted enrollment to HLA-A02:01-positive individuals (Table 1). HLA-A02:01 is the most prevalent HLA class I allele worldwide, present in over 50% of individuals across ethnicities [65], but antigen-specific T cell responses, epitope immunodominance hierarchies, and T cell receptor repertoire usage are known to vary across HLA haplotypes. The CD57^+^ exhaustion/senescence signatures in SARS-CoV-2-specific CD8^+^ T cells described here, and the broader immune phenotypes reported throughout this study, should therefore be interpreted as applying specifically to HLA-A02:01-positive individuals and may not directly generalize to individuals expressing other HLA-A, -B, or -C haplotypes. Future studies incorporating tetramers restricted to additional common HLA haplotypes (e.g., HLA-A01:01, HLA-A03:01, HLA-A24:02, HLA-B07:02) will be needed to determine whether the exhaustion–senescence axis identified here extends broadly across the SARS-CoV-2-specific T cell response or is specific to the HLA-A02:01-restricted epitopes.

An additional limitation is that, because this study was designed to focus on cellular immunity, we did not measure SARS-CoV-2-specific neutralizing antibody (Nab) titers in our SYMP and ASYMP cohorts. This omission is relevant to the interpretation of the immune phenotypes described in this study, since several studies indicate that neutralizing antibodies also diverge by disease severity [66,67,68]. Severe and critically ill patients have repeatedly been shown to mount earlier and higher-titer Nab and anti-receptor-binding-domain antibody responses than those with mild or asymptomatic infection, consistent with greater or more prolonged antigen exposure driving both humoral and cellular activation in parallel [66]. Beyond antibody titer, the qualitative features of the antibody response differ with severity. Severe and critically ill COVID-19 patients are characterized by an early enrichment of afucosylated, poorly neutralizing IgG that binds activating Fcγ receptors on myeloid cells and drives pro-inflammatory cytokine release, a signature that is absent in milder and asymptomatic COVID-19 patients and distinct from vaccine-elicited antibodies [67]. A recent 12-month longitudinal study similarly found that anti-nucleocapsid and anti-receptor-binding-domain antibody titers remained higher throughout convalescence in patients with more severe acute disease, with hospitalized individuals also showing faster antibody decay [68]. Together, these findings raise the possibility that the divergent NK and T cell phenotypes we report between SYMP and ASYMP patients occur alongside, and may be mechanistically linked to, divergent humoral responses in antibody quantity, glycosylation, and Fc-effector function. Future studies pairing cellular phenotyping with neutralizing antibody titers and Fc-glycan profiling will be needed to determine how the humoral and cellular arms of immunity jointly shape COVID-19 severity.

The convergence of checkpoint and senescence markers identified in this study points to two non-mutually exclusive therapeutic avenues. First, blockade of inhibitory receptors such as PD-1, TIM-3, and LAG-3, already in clinical use in oncology, could in principle be repurposed to restore effector function in exhausted NK and T cell populations during severe COVID-19 [69], though this strategy carries the risk of exacerbating the hyperinflammatory state that also characterizes severe disease. Second, selective clearance of CD57^+^ senescent NK cells with senolytic agents represents an alternative strategy aimed at removing dysfunctional, cytotoxic-but-unproductive NK cells rather than reactivating them [70]. Neither approach has been tested in the context of acute COVID-19, and the causal contribution of exhaustion versus senescence to disease severity, as opposed to their status as correlates, remains undefined. Future mechanistic studies, including longitudinal sampling and functional rescue experiments in checkpoint-blocked or senolytic-treated cells, will be needed to determine which of these pathways, if either, is a tractable target for intervention.

In conclusion, this study presents an in-depth analysis of NK and T cell phenotypic and functional characteristics associated with severe COVID-19 disease. These findings may help inform future investigations of immunotherapeutic strategies aimed at alleviating the symptoms of severe COVID-19.

## Figures and Tables

**Table 1:** Baseline characteristics of the HLA-A2/HLA-DR-positive SARS-CoV-2 study cohort, including age, sex, race, HLA-A*02:01 and HLA-DR distribution, and COVID-19 disease severity, presented separately for unvaccinated healthy donors, asymptomatic (ASYMP), and symptomatic (SYMP) individuals enrolled in this study.

Characteristic	Healthy (*n* = 10)	ASYMP (*n* = 10)	SYMP (*n* = 10)
Age, median (range), yr	39 (21–67)	39 (21–67)	39 (21–67)
Sex, F/M (%)	4/6 (40%/60%)	5/5 (50%/50%)	4/6 (40%/60%)
Race, Caucasian/non-Caucasian (%)	4/6 (40%/60%)	4/6 (40%/60%)	4/6 (40%/60%)
HLA-A*02:01-positive, *n* (%)	10 (100%)	10 (100%)	10 (100%)
HLA-DR-positive, *n* (%)	10 (100%)	10 (100%)	10 (100%)
COVID-19 severity (per CDC criteria)	—	None	Critical illness

## Data Availability

The original contributions presented in this study are included in the article/supplementary material. Further inquiries can be directed to the corresponding author.

## Supplementary Materials

The following supporting information can be downloaded at: https://www.mdpi.com/article/10.3390/v18090940/s1, Figure S1: Elevated Plasma levels of selective cytokines in COVID-19 ASYMP and SYMP individuals compared to Healthy controls: Cytokine expression levels of two replicates per sample were measured in plasma samples of COVID-19 ASYMP, SYMP, and Healthy individuals using Luminex. (A) Bar graphs with individual values showing the average amount of TNF-a (pg/mL) produced from ASYMP, SYMP, and Healthy individuals. (B) Bar graphs with individual values showing the average amount of IFN-y (pg/mL) produced from ASYMP, SYMP, and Healthy individuals. (C) Bar graphs with individual values show the average IL-6 (pg/mL) produced from ASYMP, SYMP, and Healthy individuals. (D) Bar graphs with individual values show the average IL-8 (pg/mL) produced from ASYMP, SYMP, and Healthy individuals. (E) Bar graphs with individual values show the average IL-17 (pg/mL) produced from ASYMP, SYMP, and Healthy individuals. Shown are the results from ten ASYMP and ten SYMP patients in 2 consecutive experiments. The indicated P values, calculated using an unpaired *t*-test, show statistical significance between SYMP and Healthy individuals.
